# Accuracy of the Apple watch for detection of AF: A multicenter experience

**DOI:** 10.1111/jce.15892

**Published:** 2023-03-30

**Authors:** Jeremiah Wasserlauf, Kelly Vogel, Cailin Whisler, Emelia Benjamin, Robert Helm, Daniel A. Steinhaus, Omair Yousuf, Rod S. Passman

**Affiliations:** 1 North Shore University Health System, Evanston, Illinois, USA; 2 Northwestern Medicine, Chicago, Illinois, USA; 3 Johns Hopkins University School of Medicine, Baltimore, Maryland, USA; 4 Boston University School of Medicine, Boston, Massachusetts, USA; 5 Saint Luke’s Cardiovascular Consultants, Kansas City, Missouri, USA; 6 Carient Heart & Vascular, Manassas, Virginia, USA

**Keywords:** atrial fibrillation, smartwatch, wearable

## Abstract

**Introduction:**

The Apple watch (AW) irregular rhythm notification (IRN) feature uses photoplethysmography to identify prolonged episodes of irregular rhythm suggestive of atrial fibrillation (AF). IRN is FDA cleared for those with no previous history of AF, however, these devices are increasingly being used for AF management. The objective of the present study was to determine the accuracy of the IRN in subjects with a previous diagnosis of nonpermanent AF.

**Methods:**

Subjects with a history of nonpermanent AF and either an insertable cardiac monitor (ICM) or cardiac implanted electronic device (CIED) with <5% ventricular pacing were fitted with an AW Series 5 for 6 months. AF episodes were compared between the ICM/CIED and IRN. The primary endpoints were sensitivity, specificity, positive predictive value (PPV), and negative predictive value (NPV) of the IRN by subject for AF ≥1 h. Secondary endpoints were sensitivity and PPV by AF episode ≥1 h. Analysis was limited to a maximum of 10 ICM/CIED episodes per subject and included only those AF episodes occurring during active AW use confirmed by activity data.

**Results:**

Thirty participants were enrolled. Mean age was 65.4 ± 12.2 years and 40% were female. There were 10 ICMs and 20 CIEDs. Eleven subjects had AF on ICM/ CIED while the AW was worn, of whom 8 were detected by IRN. There were no false positive IRN detections by subject (“by subject” 72% sensitivity, 100% specificity, 100% PPV, and 90% NPV). Five subjects had AF only when the AW was not worn. There were a total of 70 AF episodes on ICM/CIED, 35 of which occurred while the AW was being worn. Of these, 21 were detected by IRN with 1 false positive (“by episode” sensitivity = 60.0%, PPV = 95.5%).

**Conclusion:**

In a population with known AF, the AW IRN had a low rate of false positive detections and high specificity. Sensitivity for detection by subject and by AF episode was lower. The current IRN algorithm appears accurate for AF screening as currently cleared, but increased sensitivity and wear times would be necessary for disease management.

## 1 | INTRODUCTION

**FIGURE 1:**
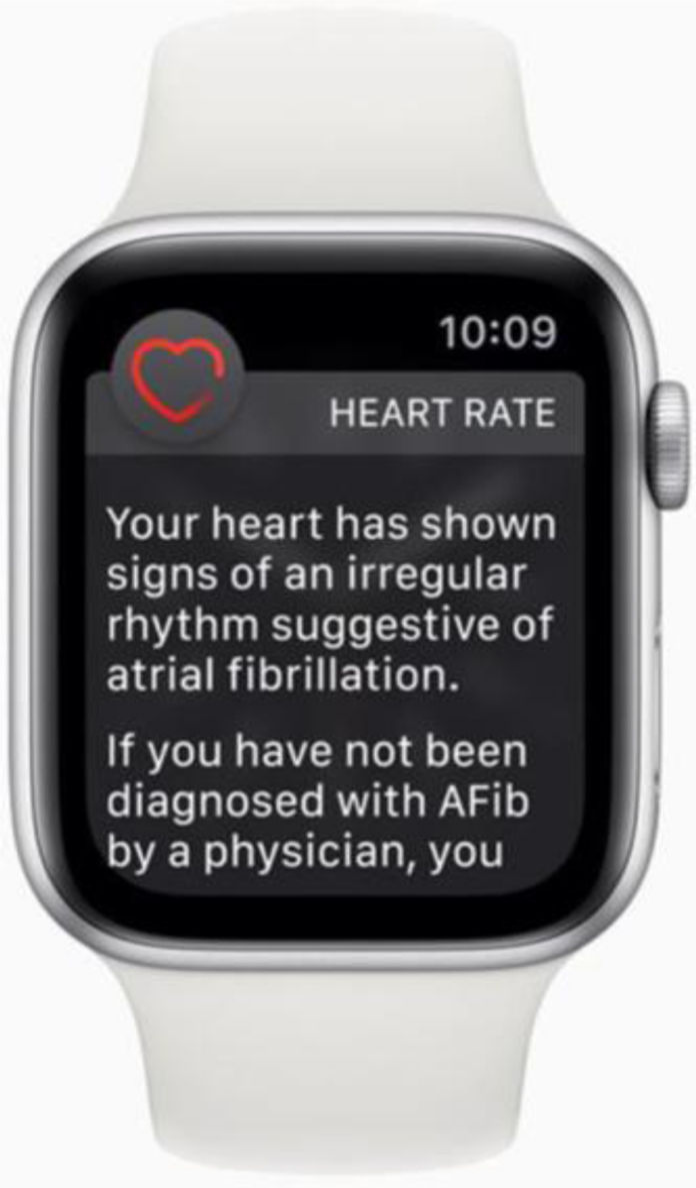
Example of an irregular rhythm notification.

Atrial fibrillation (AF) is the most common sustained arrhythmia in adults, has a lifetime risk of 25%−33%, and is associated with heart failure, stroke, dementia, and death.^1,2^ Episodes of AF can be asymptomatic, and therefore modalities for continuous monitoring may be used to quantify the occurrence of AF over a prolonged timeframe. The Apple watch (AW) irregular rhythm notification (IRN) feature uses photoplethysmography to identify prolonged episodes of irregular rhythm suggestive of AF (Figure 1). IRN is FDA cleared for those with no previous history of AF, however, these devices are increasingly being used for AF management. The objective of the present study was to determine the accuracy of the IRN for AF detection in subjects with a previous diagnosis of nonpermanent AF.

## 2 | METHODS

### 2.1 | Study design and enrollment

Thirty patients were enrolled in this study from three hospital systems (Northwestern Medicine; St. Luke’s Health System; and Boston Medical Center) between January 2020 and October 2021. Inclusion criteria consisted of a previously implanted insertable cardiac monitor (ICM) or cardiac implanted electronic device (CIED), AF lasting ≥1 h on a device tracing within 90 days before enrollment, and possession of an iPhone compatible with an AW Series 5 or later (iPhone 5s or later with iOS 12 or later). Exclusion criteria included permanent AF, >5% ventricular pacing in those with CIEDs, tattoo on the wrist where the watch was to be worn, and prior surgery on the ipsilateral radial artery.

Demographic and baseline characteristics were collected for each patient by way of medical record review and a participant interview. Participants were fitted with an AW Series 5 and asked to wear the watch during waking hours for a minimum of 14 h per day. Participants were permitted to keep the AW at the conclusion of the study.

The study was compliant with the Declaration of Helsinki, and all patients provided informed consent. The study was approved by the Institutional Review Boards of all three participating centers. Apple was not involved in any aspect of the study.

### 2.2 | Data collection and analysis

The criteria used by the AW to generate an IRN have been previously published by the device manufacturer.^3^ Briefly, the AW records the interval of time between heartbeats known as a tachogram every 2−4 h at baseline. If the rhythm is classified as irregular by the device algorithm, then the frequency of tachogram collection is increased to occur at 15 min intervals. If five out of six sequential tachograms are classified as irregular within a 48 h period then an IRN is generated. If there are two consecutive tachograms classified as regular then tachogram collection is reset to the baseline frequency of every 2−4 h. The AW collects tachograms only if the user is still enough to obtain a recording.^3^

Participants provided screen shots of all IRNs received during the study period and these episodes were compared to downloads from the patient’s ICMs/CIEDs. AF events detected by ICM/CIED without an associated IRN were further investigated by a screenshot of the participant’s “Activity” page for the corresponding day. If the participant’s “Move” bar graph was at zero during the time that the ICM/CIED detected an event, the participant was deemed to not be wearing their AW during this time.

The primary endpoints were sensitivity, specificity, positive predictive value (PPV), and negative predictive value (NPV) of the IRN per subject for detection of AF lasting 1 h or longer while the AW was worn. Subjects with at least one true positive AF detection during AW wear time were considered a true positive subject. The secondary endpoints were sensitivity and PPV of the IRN by AF episode lasting 1 h or longer while the AW was worn. In this episode‐based analysis, all episodes were counted equally, irrespec- tive of the subject who had the episode. If continuous AF developed during the study, this was considered to be a single episode. Only those ICM/CIED events that occurred while the smartwatch was simultaneously being worn were included in the primary analysis. Any ICM/CIED‐detected AF episode lasting ≥1 h that overlapped either all or in part with IRN notifications was classified as a true positive AF episode. An episode of AF detected by the IRN with no detection by the ICM/CIED was classified as a false positive episode. False negative episodes were those detected by the ICM/CIED with no overlapping IRN. In contrast to true positive, false positive, and false negative episodes, the true negative episodes could not be defined in the episode‐based approach because the ICM/CIEDs do not routinely collect electrograms in sinus rhythm. Therefore, only PPV and sensitivity could be assessed whereas NPV, specificity and accuracy were undefined. A maximum of 10 episodes were included per subject. Subjects were followed for 6 months.

Statistical analysis was performed using Excel version 2208 (Microsoft). Descriptive statistics are reported as count and percent- age for categorical variables and median, mean, SD, and interquartile range for continuous variables.

## 3 | RESULTS

Thirty participants were enrolled. The mean age was 65.4 ± 12.2 years and 40% were female. Implanted devices consisted of 10 ICMs and 20 CIEDs. Baseline characteristics are shown in the Table 1. All patients had AF detected on their ICM/CIED as an enrollment criterion.

**FIGURE 2:**
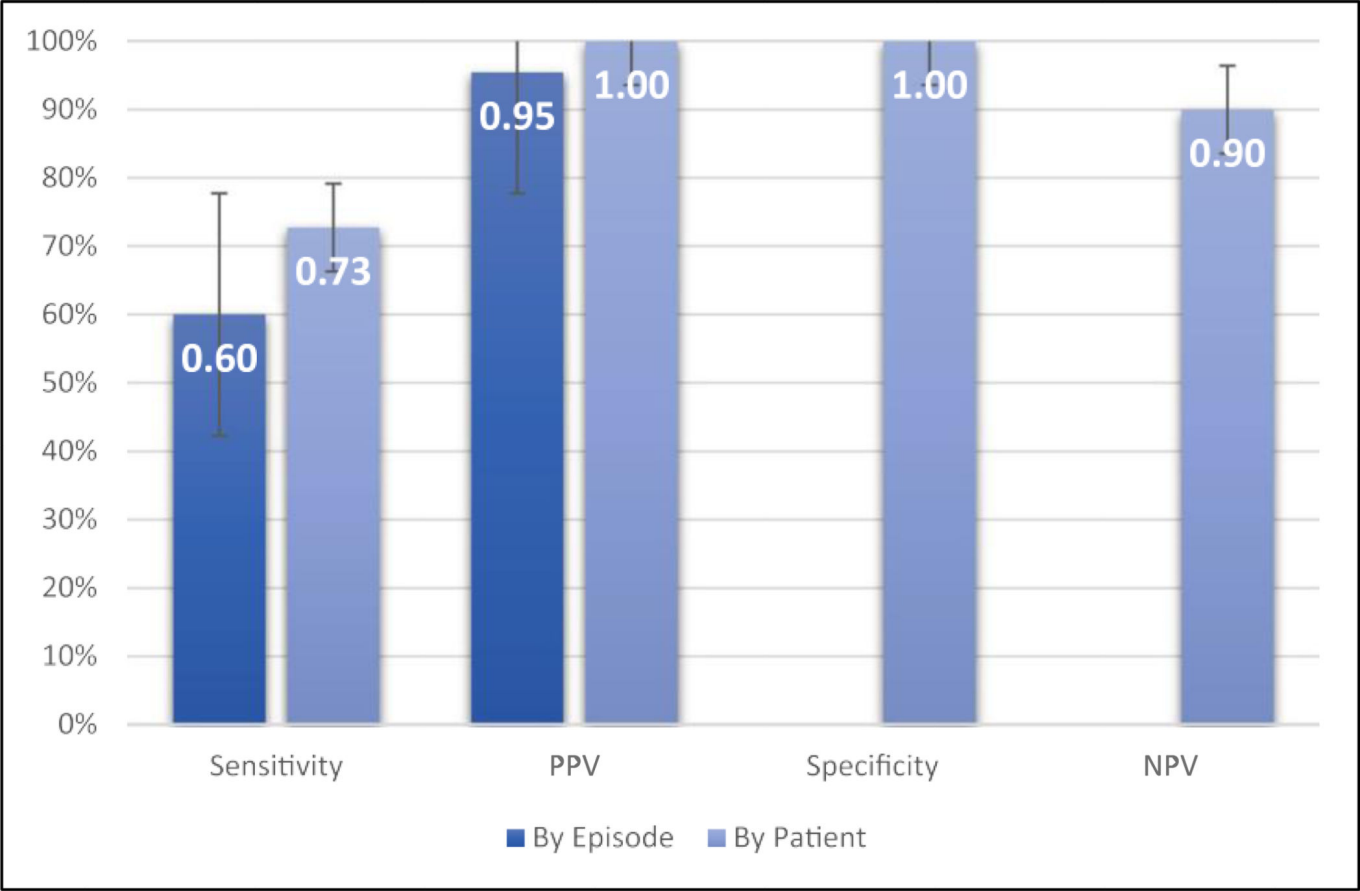
Accuracy of the IRN by episode and by patient. IRN, irregular rhythm notification; NPV, negative predictive value; PPV, positive predictive value.

Eleven subjects had AF on the ICM/CIED while the watch was worn during 6 months of follow‐up. Eight of these subjects were detected by the IRN. There were no false positive classifications by subject. Therefore, the primary endpoints of accuracy “by subject” were a sensitivity of 72%, specificity of 100%, PPV of 100%, and NPV of 90%. Five subjects had AF only when the watch was not worn. Results are shown in Figure 2.

**FIGURE 3:**
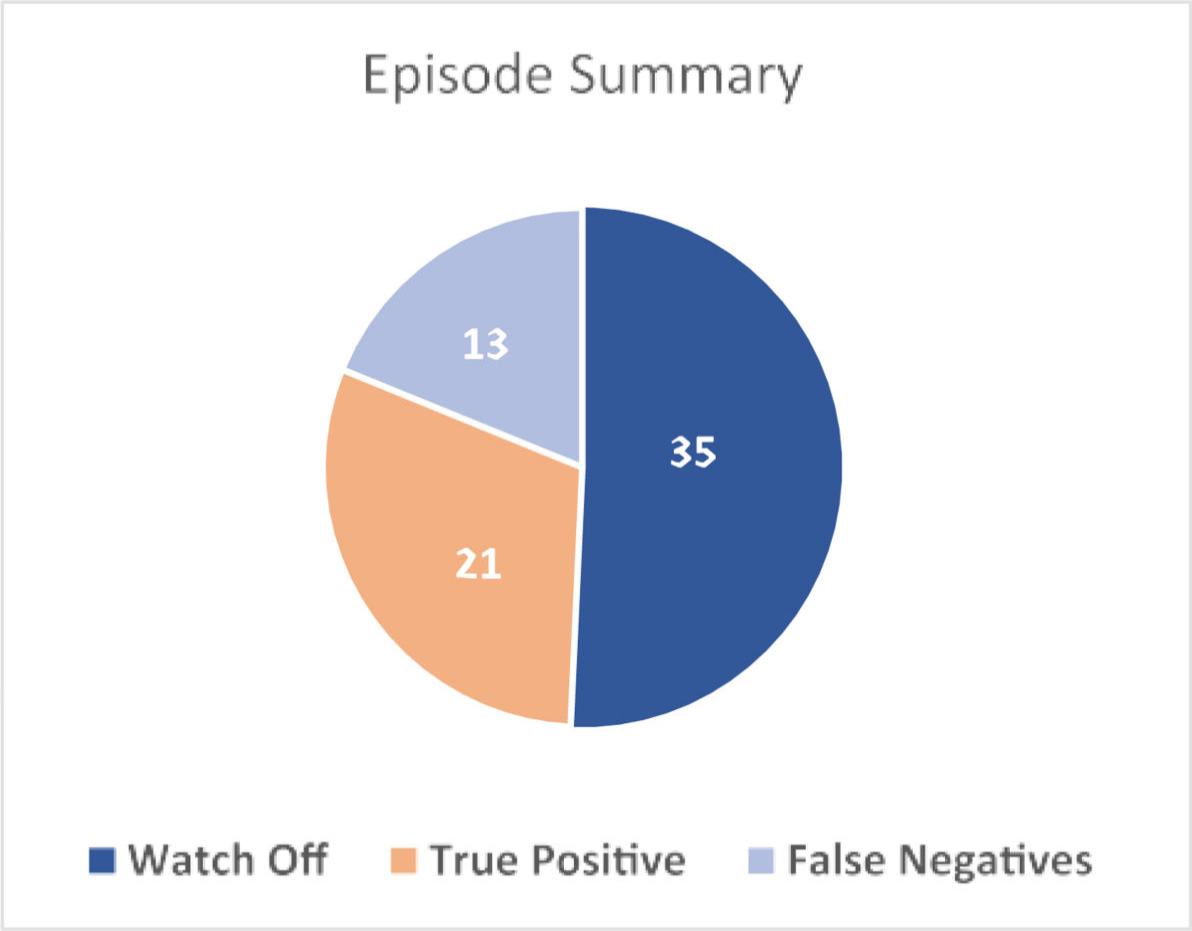
Summary of AF episodes by detection. AF, atrial fibrillation.

There were a total of 70 AF episodes on ICM/CIED, 35 of which occurred while the watch was being worn. Of these, 21 were detected by IRN with 1 false positive (Figure 3). The secondary endpoints of accuracy “by episode” during watch wear time were, therefore, a sensitivity of 60.0% and a PPV of 95.5% (Figure 2) for episodes lasting 1 h or longer. The sensitivity for AF episodes lasting 1−12 and >12 h was 58% and 75%, respectively (*p* = .635). The duration of missed episodes is provided in the Supporting Information.

## 4 | DISCUSSION

In the present study, the AW IRN feature was associated with a low rate of false positive detections but only modest sensitivity for detection of AF in a population with a previously documented history of the condition.

Previous research on the IRN has focused on accuracy for AF detection in a screening population. The Apple Heart Study recruited over 419 000 subjects without a patient‐reported history of AF to be monitored using the IRN.^4^ There were 2161 subjects (0.52%) who received a notification. An ECG patch was mailed to these subjects and used as a benchmark to assess the accuracy for subsequent IRNs during concurrent patch monitoring. Four hundred and fifty subjects returned their ECG patch with analyzable data, and in the 86 participants who received an IRN during concurrent use of the ECG patch, 72 had AF detected with the ECG patch resulting in a PPV was 0.84. A similar large scale study conducted on the Fitbit irregular heart rhythm detection feature resulted in a PPV of 98.2%,^5^ and the Huawei Heart Study reported a PPV of 91.6%.^6^

While these data support the FDA‐labeled indication of the IRN in a screening population without known AF, IRN is increasingly being used for management of patients with an existing history of AF. A prior study by our group assessed the accuracy of a different AF sensing watch compared to simultaneous recordings from an ICM.^7^ The AF sensing watch consisted of an AW Series 2, a watch band with ECG sensor (KardiaBand; AliveCor) and an experimental app with a deep learning algorithm. The findings were 95.7% sensitivity for AF episodes and 100% sensitivity for subjects with AF lasting 1 h or more while the watch was worn, as well as 39.9% PPV for AF episodes. Differences in algorithms to explain the lower sensitivity and higher PPV of the present study are proprietary, but may, in part, relate to tachogram collection by the AW which is limited to 15 min intervals when the user is still. By contrast, the AF sensing watch from the previous study acquired heart rate measurements every 5 s regardless of activity. The “AFib History” feature included with the more recent AW “watchOS 9” operating system provides an estimate of AF burden. This feature was not tested in the present study and may perform differently than IRN.

There are several limitations to the present study. First, the AW used in this study has a battery life of approximately 18 h and must be removed for charging, thereby not detecting episodes of AF occurring during this time. As described above, tachogram collection is limited to periods when the user is still, and future advances that would permit tachogram collection during activity would be expected to improve sensitivity. An AF threshold of ≥1 h was used for inclusion of AF episodes in light of several studies suggesting that shorter episodes are associated with higher FP rates on ICMs.^8,9^ Given that 5 out of 6 tachograms spaced 15 min apart are necessary for an IRN, it is conceivable that a minimum of 75 min as opposed to 60 min of AF are needed to trigger an alert. Our data show that only 2 episodes of AF lasting between 60 and 75 min occurred while the watch was being worn and that the device did alert for 1 of those episodes. Excluding the 2 episodes of 60−75 min did not change the overall sensitivity. The IRN algorithm would not, by design, detect AF episodes shorter than this. The study evaluated a small sample size of 30 subjects from three referral center populations which may limit generalizability. In addition, absorption of light from the green LED on the AW may vary depending on skin tone. The AW adjusts light output and sensitivity for different skin types and tones. Internal validation performed by the manufacturer was reported to have no significant difference in sensitivity or specificity across skin types or tones including nearly 5% of enrolled subjects with Fitzpatrick type VI skin.^3^

## 5 | CONCLUSIONS

In a population with known AF, the AW IRN had a low rate of false positive detections and high specificity for subjects with AF. Sensitivity for detection by subject and by AF episode was lower. The current IRN algorithm appears accurate for AF screening as currently indicated, but increased sensitivity and wear times may be necessary for disease management.

## Supplementary Material

Supplemental Table - Duration of Missed Episodes

## Figures and Tables

**TABLE 1:** Characteristics of patients enrolled.

Characteristic	Mean (SD) or *n* (%)
Age, years	65.4 (12.2)
Sex	
Female	12 (40%)
Male	18 (60%)
Race	
White	27 (90%)
Black	1 (3%)
Asian	1 (3%)
Declined	1 (3%)
Ethnicity	
Non-Hispanic	28 (93%)
Hispanic	1 (3%)
Declined	1 (3%)
Device	
CIED	20 (67%)
ICM	10 (33%)

Abbreviations: CIED, cardiac implanted electronic device; ICM, insertable cardiac monitor.

## Data Availability

Data supporting the results in the paper will be provided upon request.
