# Supplementary material for: Accuracy of the Apple watch for detection of AF: A multicenter experience
Source: J Cardiovasc Electrophysiol. Author manuscript; Available in PMC 2025 Jan 2. (PMC11694482; doi:10.1111/jce.15892)
Supplement: Supplemental Table - Duration of Missed Episodes [file NIHMS2032953-supplement-Supplemental_Table_-_Duration_of_Missed_Episodes.docx]

| Duration of Missed Episodes |
| --- |
| 1:00:00 |
| 1:16:00 |
| 1:26:00 |
| 2:10:00 |
| 3:34:00 |
| 3:46:00 |
| 4:02:00 |
| 4:15:19 |
| 5:04:50 |
| 5:56:00 |
| 6:58:00 |
| 9:24:00 |
| 10:08:00 |
| 18:14:09 |

Supplemental table
